# Mixotrophic Growth of *Chlorella sorokiniana* on Acetate and Butyrate: Interplay Between Substrate, C:N Ratio and pH

**DOI:** 10.3389/fmicb.2021.703614

**Published:** 2021-07-02

**Authors:** Julien Lacroux, Jordan Seira, Eric Trably, Nicolas Bernet, Jean-Philippe Steyer, Robert van Lis

**Affiliations:** Laboratoire de Biotechnologie de l’Environnement, Institut National de la Recherche Agronomique, Université de Montpellier, Narbonne, France

**Keywords:** microalgal growth, mixotrophic cultivation, dark fermentation, volatile fatty acids, microalgal lipids, pH

## Abstract

Microalgae can be cultivated on waste dark fermentation effluents containing volatile fatty acids (VFA) such as acetate or butyrate. These VFA can however inhibit microalgae growth at concentrations above 0.5-1 g_C_.L^–1^. This study used the model strain *Chlorella sorokiniana* to investigate the effects of acetate or butyrate concentration on biomass growth rates and yields alongside C:N:P ratios and pH control. Decreasing undissociated acid levels by raising the initial pH to 8.0 allowed growth without inhibition up to 5 g_C_.L^–1^ VFAs. However, VFA concentration strongly affected biomass yields irrespective of pH control or C:N:P ratios. Biomass yields on 1.0 g_C_.L^–1^ acetate were around 1.3-1.5 g_C_.g_C_^–1^ but decreased by 26-48% when increasing initial acetate to 2.0 g_C_.L^–1^. This was also observed for butyrate with yields decreasing up to 25%. This decrease in yield in suggested to be due to the prevalence of heterotrophic metabolism at high organic acid concentration, which reduced the amount of carbon fixed by autotrophy. Finally, the effects of C:N:P on biomass, lipids and carbohydrates production dynamics were assessed using a mixture of both substrates. In nutrient replete conditions, *C. sorokiniana* accumulated up to 20.5% carbohydrates and 16.4% lipids while nutrient limitation triggered carbohydrates accumulation up to 45.3%.

## Highlights

- *Chlorella sorokiniana* tolerates acetate and butyrate concentrations up to 5 g_C_.L^–1^.
- Biomass yields were correlated to initial substrate concentration.
- pH stabilization is mandatory for optimum growth on acetate or butyrate.
- *Chlorella sorokiniana* accumulates carbohydrates in short term nutrient limitation.

## Introduction

Dark fermentation (DF) has gained interest over the past 20 years since it enables waste treatment alongside hydrogen (H_2_) production, which is projected to be a sustainable vector for the transportation sector (Hosseini and Wahid, 2016). DF corresponds to the acidogenic stage of the anaerobic digestion process that ultimately leads to the production of methane. However, bioH_2_ production by fermentative bacteria is limited by their metabolic constraints: the degradation of organic material into H_2_ is incomplete with a theoretical maximum yield of only 33% of the initial chemical oxygen demand (COD). The remaining COD is retrieved in the form of various soluble metabolites, such as VFA. DF mainly leads to the production of acetate and butyrate in variable proportions according to the waste composition, with an average value of 0.17 g_Acetate_.g_COD_^–1^ and 0.675 g_Butyrate_.g_COD_^–1^ (Moscoviz et al., 2018). The production of such molecules represents opportunities for the bio-economy, as they could serve as cheap organic substrates for other processes (Guwy et al., 2011). More particularly, the mixotrophic growth of microalgae on fermentative metabolites has been recently investigated for the production of lipids (Ren et al., 2018). Mixotrophic growth occurs when microalgae grow by simultaneously using inorganic carbon (CO_2_) using photosynthesis as well as organic carbon sources (Chew et al., 2018). This cultivation mode allows increasing productivity compared to pure autotrophy thanks to the heterotrophic metabolism as well as reduction in CO_2_ emission compared to pure heterotrophy thanks to photosynthesis (Smith et al., 2015). The coupling is however limited partly by the low VFA concentration that microalgae can tolerate. For example, Fei et al. (2015) indicated that *C. protothecoïdes* could not grow on a mixture of VFA composed of 1.6 g_C_.L^–1^ acetate, 0.5 g_C_.L^–1^ butyrate and 1 g_C_.L^–1^ propionate. Mixotrophic growth of *C. sorokiniana* on concentrations above 4 g_C_.L^–1^ (10 g.L^–1^) acetate was reported, but with a prolonged lag phase (Chen et al., 2017). Although the VFA concentrations can be lowered by diluting the DF effluent (DFE), this would result in reduced final biomass concentration, increasing the harvesting costs. An alternative is to increase the proportion of acetate in DFE to promote microalgae growth (Fei et al., 2015; Baroukh et al., 2017), resulting in an acetate:butyrate (A:B) mass ratio equal or above 1. This composition is however unrepresentative of an average DFE composition (Moscoviz et al., 2018). As such, finding conditions allowing microalgal growth on concentrated butyrate remains a necessary step to improve the coupled DF-microalgae process. In most studies, microalgae are cultivated at a pH between 6.5 and 7.0. The pKa of both acetate and butyrate is close to 4.8. Since microalgae inhibition by VFA is mainly related to the undissociated acid concentration (Lacroux et al., 2020), it is suspected that raising initial pH to minimize the undissociated acid concentration may allow microalgal growth at higher than previously reported acetate or butyrate concentrations. Moreover, if acetate has been consistently reported to promote growth of many green algae (Perez-Garcia et al., 2011), butyrate conversion represents another bottleneck. The few studies reporting the physiology of *Chlorella* and related species on butyrate or mixtures of VFA in axenic conditions were carried out in heterotrophy at butyrate concentrations lower than 0.5 g_C_.L^–1^ due to its inhibitory effect (Liu et al., 2013a; Turon et al., 2015a). Thus, gathering knowledge about the effect of higher butyrate concentration on algal physiology in axenic mixotrophic condition remains necessary to better understand microalgae behavior in real processes.

Besides organic carbon, DFEs also contain mineral nutrients (N and P) in the form of NH_4_^+^ and PO_4_^3–^ as a result of protein mineralization. These nutrients are directly usable by microalgae but their amounts relative to carbon may impact the performances of the microalgae cultivation. Some studies report DFE mass C:N:P ratios of 317:24:1 (Cho et al., 2015), 73:5.6:1 (Cho et al., 2017), and 10:0.7:1 (Qi et al., 2018). In these studies, the C:N ratio seems relatively stable between 13 and14 while the P amount greatly varies. As reference, an average algal biomass molar C:N:P is close to 106:16:1 (Redfield Ratio Trends, 2000). The standard C:N ratio is thus 6.6 even though biomass C:N ratios were reported at 5.6 for *C. sorokiniana* (Kumar et al., 2014) or 7.1 for *C. reinhardtii* (Boyle and Morgan, 2009) growing mixotrophically on acetate. Higher C:N are thus indicative of a N-deficiency, which will induce carbon reserve formation mostly as lipids and sugars (Sajjadi et al., 2018). However, if acetate was shown to increase carbon reserves compared to autotrophy (Park et al., 2012), the effect of butyrate on accumulation products has not been specifically studied.

**FIGURE 1:**
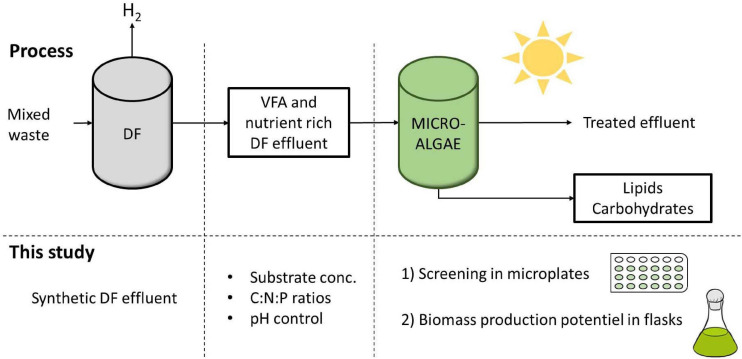
Integration of mixotrophic microalgae cultivation with dark fermentation (DF) for the concomitant production of energy as H_2_ and lipids and effluent treatment. In this study, several parameters were first screened in microplates and biomass production potential of *C. sorokiniana* was further evaluated in flasks.

This study aimed at providing new insights into the effect of high substrate concentration, as well as the combined effect of pH variations and C:N:P ratios on *C. sorokiniana* mixotrophic growth, both in terms of biomass yield and intracellular carbon reserve accumulation (Figure 1). *C. sorokiniana* was chosen as a model microalgae strain due to its ability to consume butyrate faster than other green strains (Lacroux et al., 2020). First, a microplate cultivation protocol was designed and compared to flask cultivation. The microplate protocol enabled to study the effect of multiple parameters on the growth of *Chlorella sorokiniana*, such as acetate and butyrate at different concentrations (0.5 to 5 g_C_.L^–1^ each), various buffer concentrations and C:N:P ratios. Finally, in order to evaluate the biomass production potential of *C. sorokiniana*, cultures in Erlenmeyer flasks were carried out on synthetic DFE. Dynamics of the biomass production, VFA uptake, pH and biomass characteristics of *C. sorokiniana* growing on synthetic were assessed.

## Materials and Methods

### Strain and Standard Culture Media

The algal strain used is a *Chlorella sorokiniana* lab strain, obtained from the SAG culture collection (Goettingen, Germany) with number SAG 211-8k. It was maintained on a synthetic medium referred to as HAP, which is based on Tris-Acetate-Phosphate (TAP) medium using HEPES buffer instead of Tris. Buffer concentration was set to 20 mM unless stated otherwise. Acetate or butyrate were added as sodium salts at 0.5 g_C_.L^–1^ for pre-cultures. When butyrate was used, the medium is referred to as HBP. Beijerincks solution was added at 25 mL.L^–1^ leading to an ammonium (NH_4_^+^) concentration of 7.5 mM, 0.6 mM of MgSO_4_ and 0.3 mM of CaCl_2_. Phosphorus (K_2_HPO_4_) was added at 1 mM and 1 mL.L^–1^ of Hutner’s trace elements was also supplemented. The 1000X Hutner’s solution was prepared by dissolving H_3_BO_3_ (0.6 g), CuSO_4_.5H_2_O (0.25 g), NaFeEDTA (7.5 g), Na_2_EDTA (15.0 g), MnCl_2_.4 H_2_O (2.0 g), NaMO_4_.2H_2_O (0.25 g), ZnSO_4_.7H_2_O (1.2 g) and CoCl_2_.6H_2_O (0.25 g) in 1L distilled water. Medium pH was adjusted to 8.0 by addition of NaOH prior to sterilization at 121°C for 20 min. After cooling, 100 μL.L^–1^ of vitamin B1 50 mM, biotin 1 mM and cyanocobalamin 1 mM, sterilized by filtration over a 0.2 μm filter, were added into the medium.

### Variable Culture Media in Microplates

Several media with variable substrate nature and concentrations, C:N:P ratio and buffer capacity were prepared. First, to investigate the effect of substrate concentration, concentrations of acetate or butyrate individually were varied between 0.5, 1.0, 2.0, 3.0, 4.0, and 5.0 g_C_.L^–1^. A fixed amount of 7.5 mmol N.L^–1^ (105 mg N-NH_4_.L^–1^) and of 1 mmol P.L^–1^ (15 mg P-PO_4_.L^–1^) was added to the media. Molar C:N:P varied thus from 42:7.5:1 to 416:7.5:1. Buffer capacity was set to 20 mM. The detailed composition of these media is provided in Table 1. In a second set of experiments, concentrations of acetate or butyrate were set to respectively, 1.0 and 2.0 g_C_.L^–1^. Buffer capacity was varied between 0, 20, and 100 mM. C:N:P was fixed between 83:7.5:1, 106:16:1, or 167:7.5:1. To vary C:N:P, the proper amounts of NH_4_Cl and K_2_HPO_4_ stock solutions were supplemented to the media. The detailed macromolecular composition of these media is given in Table 2.

**TABLE 1:** Macro-element composition used to assess the effect of carbon concentration on growth.

C (g_C_.L^–1^)	Acetate (g.L^–1^)	Butyrate (g.L^–1^)	N-NH_4_ (mg.L^–1^)	P-PO_4_ (mg.L^–1^)	C:N:P (molar)	Buffer capacity (mM)
0.5	1.2	-	105	15	42:7.5:1	20
	-	0.8				
1	2.5	-	105	15	83:7.5:1	20
	-	1.6				
2	5	-	105	15	166:7.5:1	20
	-	3.2				
3	7.5	-	105	15	250:7.5:1	20
	-	4.8				
4	10	-	105	15	333:7.5:1	20
	-	6.4				
5	12.5	-	105	15	416:7.5:1	20
	-	8				
						

**TABLE 2:** Macro-element composition used to assess the combined effect of buffer concentration and C:N:P on growth.

C (g_C_.L^–1^)	Acetate (g.L^–1^)	Butyrate (g.L^–1^)	N-NH_4_ (mgl.L^–1^)	P-PO_4_ (mgl.L^–1^)	C:N:P (molar)	Buffer capacity (mM)
1	2.5	-	105	15	83:7.5:1	0
	-	1.6				
1	2.5	-	175	15	106:16:1	0
	-	1.6				
1	2.5	-	105	15	83:7.5:1	20
	-	1.6				
1	2.5	-	175	15	106:16:1	20
	-	1.6				
1	2.5	-	105	15	83:7.5:1	100
	-	1.6				
1	2.5	-	175	15	106:16:1	100
	-	1.6				
2	5	-	105	15	166:7.5:1	0
	-	3.2				
2	5	-	350	30	106:16:1	0
	-	3.2				
2	5	-	105	15	166:7.5:1	20
	-	3.2				
2	5	-	350	30	106:16:1	20
	-	3.2				
2	5	-	105	15	166:7.5:1	100
	-	3.2				
2	5	-	350	30	106:16:1	100
	-	3.2				

### Cultivation on Synthetic DF Effluent

To evaluate the biomass production potential of *C. sorokiniana*, the strain was cultivated in Erlenmeyer flasks using two synthetic DFEs. The media used were based on HAP medium with modifications in the type and concentration of substrate, C:N:P and buffer concentration. VFA concentration was set to 3 g_C_.L^–1^ by mixing 1 g_C_.L^–1^ acetate and 2 g_C_.L^–1^ butyrate. This resulted in an acetate:butyrate carbon mass ratio of 1:2. This concentrations and ratio were considered as representative of an average DFE as reported elsewhere (Moscoviz et al., 2018). A medium with molar C:N:P of 106:16:1 (N-NH_4_ = 525 mg.L^–1^ and P-PO_4_ = 45 mg.L^–1^) and a buffer capacity of 100 mM was compared to a condition where pH was weakly buffered (20 mM HEPES) and nutrients limiting (250:7.5:1) (N-NH_4_ = 7.5 mg.L^–1^ and P-PO_4_ = 15 mg.L^–1^).

### Culture Conditions

The two first experiments were tested in sterile 24-well microplates. The microplate protocol is detailed in the Supplementary Material. In brief, microplates were wrapped into plastic sachets, which drastically reduced water evaporation while allowing light to penetrate. Each well was homogenized by pipetting before measurement and condensation was removed via aseptic wiping. Wells were filled with 1 mL of medium culture, leading to a headspace of 1.5 mL. Wells were inoculated with 10 μL of inoculum culture (initial OD_750_ = 0.05). This inoculum was prepared by collecting pre-cultured cells on HAP medium in the exponential phase via centrifugation at 2500 rpm for 10 min followed by suspension of pellet in sterile phosphate buffered saline to a final OD_750_ around 5. Cultivations in flasks were performed in sterile 500 mL Erlenmeyer flasks filled with 200 mL medium. All cultures were carried out in triplicates under constant light using cool-white fluorescent lamps at 100 μmoles photons.m^–2^.s^–1^ at 25°C and under agitation of 120 rpm (2.5 cm orbit).

### Biomass Measurement

Biomass production in microplates was determined via the measurement of the optical density at 750 nm (OD_750_) by an Infinite Nanoquant M200 (Tecan^®^) Spectrophotometer. Dry weight (DW) was determined after filtration over a Whatman^®^ GF/C glass microfiber filter (1.2 μm) and overnight drying at 105°C. To correlate OD_750_ to biomass dry weight, calibration curves were made. For this, 200 mL cultures of HAP or HBP medium in 500 mL flasks were sampled one to three times a day and both OD_750_ and dry weight were determined. The correlation factor to calculate the dry weight from OD_750_ values measured in microplates with covers was determined to be of 2.0745 (R^2^ = 0.998) for acetate and 1.728 (R^2^ = 0.985) for butyrate. Biomass production in flasks was determined directly by DW determination.

### VFA and Nutrient Measurements

Samples from fresh cultures were immediately centrifuged, filtered over 0.2 μm cut-off filters and frozen at –20°C until analysis. The liquid samples were analyzed by either liquid or ionic chromatography. Acetate and butyrate were measured by high performance liquid chromatography (Dionex Ultimate 3000) coupled to a refractive index detector (Waters R410). HPLC analysis was performed at a flow rate of 0.7 mL/min on an Aminex HPX-87H, 300 × 7.8 mm (Bio-Rad) column at a temperature of 35°C. H_2_SO_4_ at 10 mM was used as the mobile phase under isocratic elution. Ammonium and phosphate ions were measured using a DIONEX ICS-3000 ion chromatograph with conductimetry detection. A NG1-2 pre-column was used to avoid contamination. The eluents used for cations and anions measurements were HMSA (acid hydroxymethanesulfonic acid) (25-40 mM) at a flow rate of 0.3 mL.min^–1^, and KOH (10-74 mM) at 0.35 mL.min^–1^, respectively.

### Total Lipids and Sugars Measurements

Samples from fresh cultures were immediately centrifuged, the supernatant was discarded and the pellet stored at –20°C until analysis. Before analysis, the pellet was thawed, resuspended in distilled water and put in a 10 mL glass tubes for either lipid or sugar measurements.

Total lipids were measured by the phosphovanillin method (Mishra et al., 2014). Phosphovanillin reagent was freshly prepared before analysis by dissolving 0.6 g vanillin in 10 ml ethanol, 90 ml deionized water and 400 ml of H_3_PO_4_ (85%). The resulting reagent was stored in the dark until use. First, 2 mL of H_2_SO_4_ (98%) were added in the tubes containing microalgae samples. The tubes were heated 10 min at 100°C. After cooling on ice, the reaction was initiated by addition of 5 mL of phospho-vanillin reagent prior incubation for 15 min at 37° C. Tubes were periodically shaken by inversion. After cooling, absorbance of suspensions was measured at 530 nm with an Aqualytic^®^ spectrometer and compared to distilled water. Calibration curves were obtained using canola oil.

Total sugars were measured by the anthrone method. Anthrone reagent was prepared by dissolving 200 mg of anthrone in 100 mL of H_2_SO_4_ (98%). Two mL of anthrone reagent were added in the tubes containing microalgae. Tubes were cooled down on ice and then incubated at 100° C for 10 min. After cooling, absorbance of suspensions was measured at 625 nm with an Aqualytic^®^ spectrometer and compared to distilled water. Calibration curves were obtained using glucose solution.

### Specific Growth Rate, Biomass Yield, and Mass Balance Calculations

The specific growth rate *μ* (d^–1^) was determined using the slope of the linearized biomass concentration curve over time. At least 4 points were used for the linearization, with R^2^ values between 0.97 and 0.99.

Biomass yields *Y* (g_*CX.*_g_CS_^–1^) were calculated according to eq. (1):

(1) $$Y=\frac{X_{f}-X_{0}}{S_{0}-S_{f}}$$

where *X*_*f*_ and *X*_0_ are the biomass concentrations (g_*CX*_.L^–1^) and *S*_*f*_ and *S*_0_ are substrate concentrations (g_*CS*_.L^–1^) at the end (*t*_*f*_) or at the beginning (*t*_0_) of the cultivation phase. The amount of carbon in the biomass was estimated using the biomass molecular formula of *C. reinhardtii* cultivated in mixotrophy on acetate CH_1.62_O_0.41_N_0.14_P_0.011_ (Boyle and Morgan, 2009). In case of mixtures of acetate and butyrate, the concentration of each substrate was separately considered.

Productivities or consumption rates *Q*_*i*_ (g.L^–1^.d^–1^) were calculated according to eq. (2):

(2) $$Q_{i}=\frac{i_{f}-i_{0}}{t_{f}-t_{0}}$$

where *i*_*f*_ and *i*_0_ are the product concentrations at the end or at the beginning of the cultivation.

The mass balance was performed using eqs. (3) for acetate or (4) for butyrate.

(3) $$\alpha \,C\,H_{3}\,C\,O\,O+\beta \,N\,H_{4}+\gamma \,H_{2}\,P\,O_{4}+\varepsilon \,O_{2}+\zeta \,C\,O_{2}=\chi \,C\,H_{1.62}\,O_{0.41}\,N_{0.14}\,P_{0.011}+\lambda \,H_{2}\,O$$

(4) $$\alpha \,C\,H_{3}\,C\,H_{2}\,C\,H_{2}\,C\,O\,O+\beta \,N\,H_{4}+\gamma \,H_{2}\,P\,O_{4}+\varepsilon \,O_{2}+\zeta \,C\,O_{2}=\chi \,C\,H_{1.62}\,O_{0.41}\,N_{0.14}\,P_{0.011}+\lambda \,H_{2}\,O$$

Each stoichiometric coefficient was normalized by the amount of biomass produced.

Statistical analyses (ANOVA) were performed using GraphPad Prism V 8.0.2.

## Results and Discussion

### Microplate Protocol Validation

**FIGURE 2:**
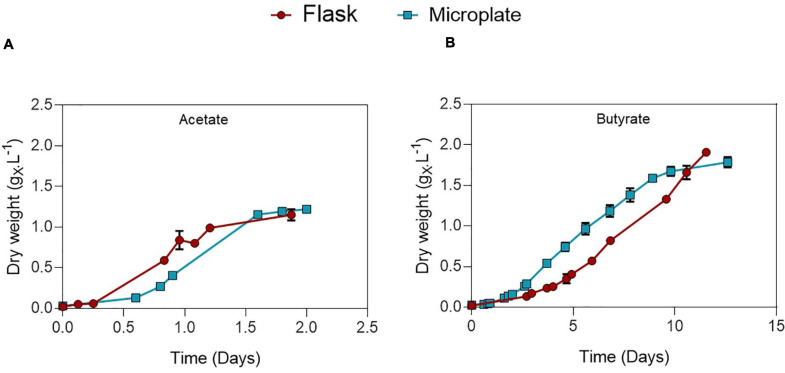
Comparison between growth of *C. sorokiniana* on 0.5 g_C_.L^–1^ acetate **(A)** or butyrate **(B)** growing in either microplate (blue) or Erlenmeyer flasks (red). Each point represents a mean ± standard deviation of 3 biological replicates.

Microplate cultivation provides an efficient way to screen multiple parameters at once and gather biomass growth dynamics data. Factors such as water evaporation, water condensation on the lids, poor mixing and optical density saturation need to be addressed to ensure accuracy of measurements. Several methods were used to properly deal with these issues, which are described in detail in the Supplementary Information. To validate this protocol and allow comparison between the microplate and flask setup, *C. sorokiniana* was cultivated on either 0.5 g_C_.L^–1^ acetate (Figure 2A) or butyrate (Figure 2B) in both microplates and Erlenmeyer flasks. The derived growth rates and final biomass concentrations, determined by gravimetric method in the case of flasks, or via correlation in the case of microplates, are given in Table 3. No significant difference in growth rates for acetate and butyrate was observed between microplates or flasks. Only a small significant increase of 6.2% was observed in final biomass concentration for butyrate in flask cultures as compared to microplates. No significant difference was observed in final biomass concentration for cells grown on acetate.,It was concluded that, although not exempt of small bias, this setup allows accurate predictions of biomass growth. The microplate protocol was thus a useful tool to screen several growth parameters at the same time.

**TABLE 3:** Growth characteristics of *C. sorokiniana* growing on 0.5 g_C_.L^–1^ acetate or butyrate in either microplate or flask.

	Acetate		Butyrate	
	μ (d^–1^)	X_max_ (g_X_.L^–1^)	μ (d^–1^)	X_max_ (g_X_.L^–1^)
Microplate	3.6 ± 0.04	1.21 ± 0.02	0.44 ± 0.02	1.79 ± 0.06^a^
Flask	3.6 ± 0.50	1.15 ± 0.07	0.41 ± 0.02	1.91 ± 0.04^b^

*X_max_: maximum biomass (g_*X*_.L^–1^), *μ*: growth rate (d^–1^). Values are given as mean ± standard deviation, *n = 3*. Letters indicate significant difference between means (*p*-value < 0.05).*

### Absence of Inhibition by VFAs on *C. sorokiniana* Mixotrophic Growth

**FIGURE 3:**
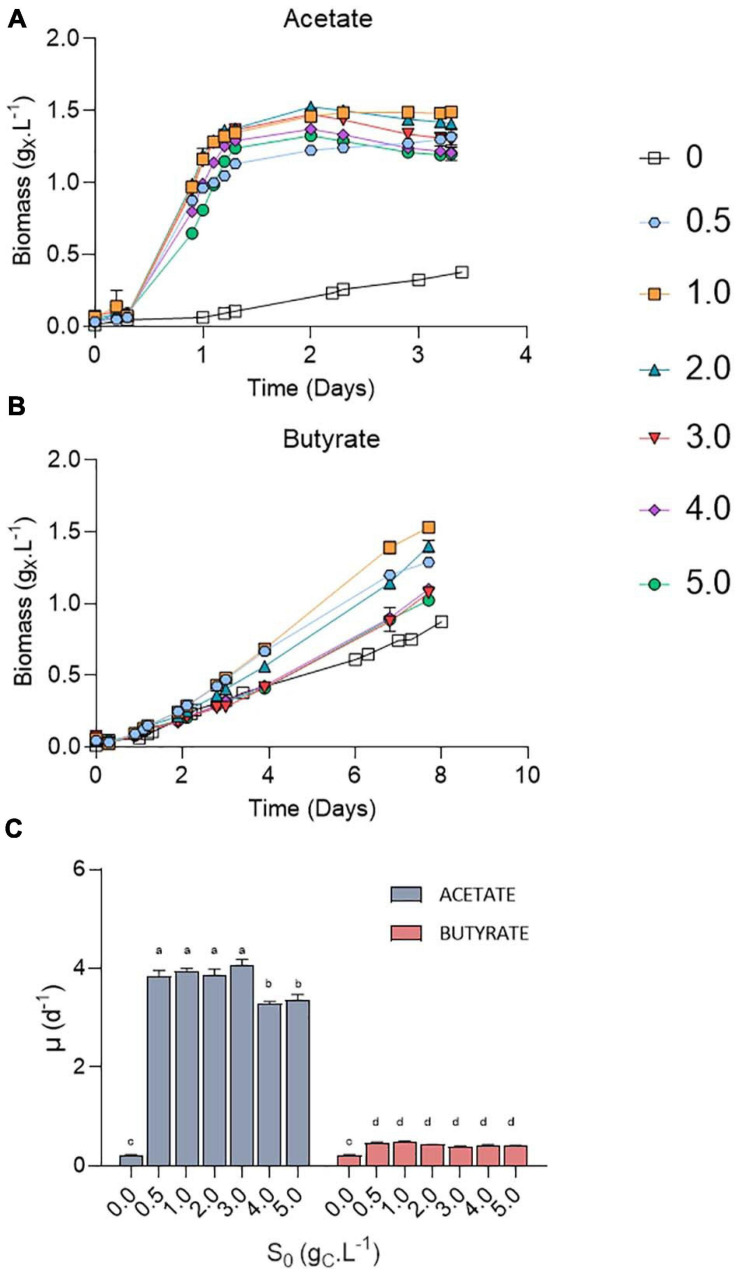
Growth curves of *C. sorokiniana* on increasing acetate **(A)** or butyrate **(B)** concentrations from 0 to 5.0 g_*CS*_.L^–1^; growth rates (μ, d^–1^) of *C. sorokiniana* on acetate or butyrate **(C)**. Growth rates on acetate were calculated using the points from 0.2 to 1.1 days, while growth rates on butyrate were based on the points from 2 to 4 days. Letters indicate significant differences between means (*p*-value < 0.05). All data are given as mean ± standard deviation of 3 biological replicates.

*Chlorella sorokiniana* was first cultivated in microplates on acetate or butyrate at concentrations ranging from 0.5 to 5.0 g_*CS*_.L^–1^ (Figure 3). An autotrophic control was also performed in the same conditions by omitting any carbon source (*S*_0_ = 0 g_*CS*_.L^–1^). Growth was observed at all tested VFA concentrations, with only a minor increase in the lag phase at the highest concentrations (Figures 3A,B). Irrespective of the organic substrate, growth rates remained stable at each concentration, indicating that no major inhibition occurred (Figure 3C). In the case of acetate, an average growth rate of 3.93 ± 0.13 d^–1^ was observed for 0.5 – 3 g_*CS*_.L^–1^ without significant difference, while increasing acetate concentration up to 4.0 – 5.0 g_*CS*_.L^–1^ resulted in a significant slight decrease to 3.33 ± 0.08 d^–1^. These values are consistent with those reported previously for mixotrophic growth of *C. sorokiniana* on acetate (4.14 ± 0.35) (Turon et al., 2015b). Use of butyrate as carbon source resulted in 10-fold lower growth rates of 0.44 ± 0.04 d^–1^ on average as compared to acetate, without significant difference between the tested concentrations. This growth rate corresponds to an average biomass productivity of 0.22 ± 0.04 g_*X*_.L^–1^.d^–1^, slightly above the productivity determined on butyrate by Turon et al. (2015b) (0.14 g_*X*_.L^–1^.d^–1^). In any case, addition of organic carbon source resulted in a significant higher growth rate compared to autotrophy (0.23 d^–1^).

Most studies dealing with the growth of microalgae on VFAs indicate substrate inhibition above concentrations in the range of few grams per liter. The main cause of microorganism growth inhibition by VFA is the accumulation of undissociated acid form (ROOH, R being the carbon chain). ROOH are lipophilic and can cross the cell membrane. Inside the cells, where pH is near neutral, the protonated form will dissociate again, leading to cytosol acidification and anion accumulation. These two combined effects can have deleterious effects on cell growth (Ricke, 2003). Although well described for bacteria or yeast (Jarboe et al., 2013), fewer studies have reported the effects of ROOH on microalgae growth. In a previous study, ROOH concentrations were found to be detrimental for microalgal growth above 13.6 mg_C_.L^–1^ butyric acid or 28.3 mg_C_.L^–1^ acetic acid (Lacroux et al., 2020). As a comparison, the concentration of acetic acid and butyric acid in the experiments conducted by Fei et al. (2015) were of 58.8 mg_C_.L^–1^ and 39.95 mg_C_.L^–1^, respectively (pH 6.3; total concentration of acetate 1.92 g_C_.L^–1^ and butyrate 1.31 g_C_.L^–1^) and led to complete growth inhibition of *C. protothecoïdes*. Inhibition can be lifted by raising inoculum density. For example, mixotrophic growth of *C. sorokiniana* on 10 g.L^–1^ (4 g_C_.L^–1^) acetate was inhibited when inoculating the medium with low cell density (OD_750_ < 0.1). The inhibition could be alleviated by raising initial cell concentration (initial OD_750_ > 1) (Van Wagenen et al., 2015). This is however not desirable since this requires high amount of initial biomass. The pH 8.0 value used here resulted in low levels (2.4 mg_C_.L^–1^ ROOH for 5 g_C_.L^–1^) of the undissociated acid form, and mixotrophic growth was thus not inhibited even using low inoculum density (initial OD_750_ = 0.05). By raising initial pH value to 8.0, it is shown here that *C. sorokiniana* can grow up to 12.5 g.L^–1^ acetate and 8.75 g.L^–1^ butyrate without major inhibition in growth rates or biomass productivities. If cultivation on high acetate concentration has already been described, this is to the best of our knowledge the first time that microalgae cultivation on pure butyrate has been successfully carried out at such concentrations.

### Decrease of Biomass Yields With Initial Organic Acid Concentration

On acetate, stationary phase was reached in 1.5 days. A maximum of 1.5 g_*X*_.L^–1^ was obtained at *S*_0_ = 1.0 g_C_.L^–1^ for both substrates. Increased *S*_0_ lead to a decrease in final biomass, from 1.4 g_*X*_.L^–1^ at 2 g_C_.L^–1^ to 1.0 (butyrate) or 1.2 (acetate) g_*X*_.L^–1^ at 5 g_C_.L^–1^. In any case, maximum biomass (*X*_*max*_) was higher than that in autotrophy, where a maximum of 0.87 g_*X*_.L^–1^ was obtained after 8 days of cultivation. It is noted that the values of *X*_*max*_ for butyrate in Figure 2B (Table 3) are higher than those in Figure 3B which can be explained by the difference in culture duration (12 days vs. 8 days).

**FIGURE 4:**
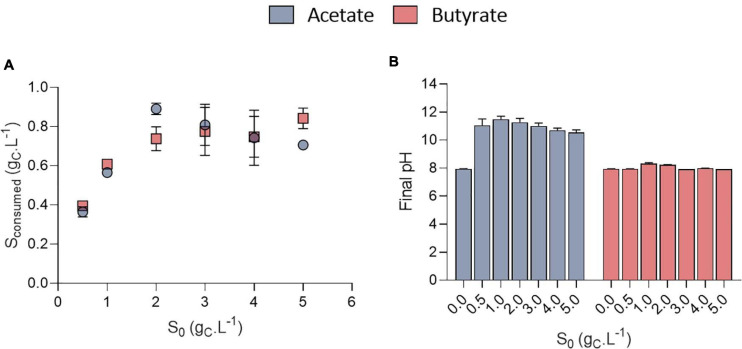
Amount of substrate consumed (g_*CS*_.L^–1^) as a function of initial substrate (S_0_, g_*CS*_.L^–1^) for acetate or butyrate, based on end point cultivation measurement (3.3 days for acetate and 7.7 days for butyrate) **(A)**; final pH measured at the end of the cultivation of *C. sorokiniana* growing on acetate or butyrate concentrations from 0 to 5.0 g_*CS*_.L^–1^ **(B)**. Data represents mean ± standard deviation of 3 biological replicates.

The growth could be attributed to organic acids consumption (Figure 4A). The amount of VFA consumed was calculated based on the difference between the amount of VFA in a well without inoculum and the inoculated ones to avoid potential biases due to VFA volatilization. Organic acids were however never completely consumed. No significant difference was observed in the amount of substrate taken up at a given *S*_0_ between acetate and butyrate. In Figure 2B, where 0.5 g_C_.L^–1^ butyrate was used, similar, values for the amount of consumed substrate were obtained as in Figure 4A (S_0_ = 0.5 g_C_.L^–1^). Since even with the extra cultivation time of the cultures of Figure 2B not all substrate was consumed, the cells likely grew only by autotrophy during these extra 4 days.

Stationary phase may have been reached either after nutrients (N or P) were depleted or due to pH elevation. Indeed, a fixed amount of 7.5 mM N-NH_4_ (105 mgN-NH_4_.L^–1^) and 1 mM P-PO_4_ (15 mgP-PO_4_.L^–1^) was added into each medium. Considering a biomass formula of CH_1.62_O_0.41_N_0.14_P_0.011_ (Boyle and Morgan, 2009) during the growth phase, the consumption of these nutrients should lead to at least the production of 0.64 g_*CX*_.L^–1^ biomass which corresponds broadly to the value obtained here (Figure 3). However, cultivation on acetate also resulted in a consistent massive pH rise at least above 10.5 (Figure 4B). It was previously reported that pH above 9.0 lowers VFA assimilation (Hwang et al., 2014; Shen et al., 2016). Consistently, it was previously shown that acetate consumption by *C. vulgaris* was doubled when controlling pH to 8.5 compared to uncontrolled condition (Yeh et al., 2012). Thus, whether organic substrate consumption stopped because of nutrient limitation or pH cannot be concluded from these experiments. Conversely, pH remained stable around 8.0 in the butyrate or autotrophic cultures. The difference in pH elevation between the two substrates could be due to the different consumption rate. As growth rate on butyrate is around five-fold lower than on acetate, atmospheric CO_2_ solubilization could compensate for the pH rise. However, this phenomenon cannot solely explain the difference observed between acetate and butyrate. Indeed, in wells without inoculum (blank), pH was seen to decrease only by 0.2 (acetate wells) or 0.4 (butyrate wells) units by the end of the cultivation period (data not shown). Alternatively, since butyrate contains twice the amount of carbon per carboxylate group assimilated by the cells, a lower pH elevation per molecule absorbed is expected. A combination of both effects, possibly alongside other unknown cellular processes, may explain the difference in pH elevation for the two substrates.

**FIGURE 5:**
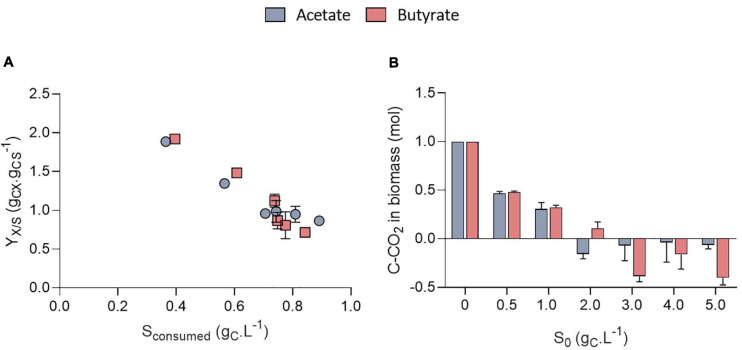
Biomass yields on substrate (Y_*X/S*_, g_*CX*_.g_*CS*_^–1^) obtained on acetate or butyrate as a function of the amount of substrate consumed **(A)**; Stoichiometric coefficient for CO_2_ fixation (C-CO_2_, mol) normalized by the amount of biomass obtained after mass balance analysis for acetate or butyrate **(B)**. All data corresponds to the final cultivation time point. All data are given as mean ± standard deviation of 3 biological replicates.

Surprisingly, the amount of consumed organic carbon was not the maximum achievable when cells were grown at initial concentrations of 0.5 g_*CS*_.L^–1^ and 1.0 g_*CS*_.L^–1^. Indeed, only 0.38 and 0.57 g_*CS*_.L^–1^ were consumed in these conditions, respectively, while up to 0.8 g_*CS*_.L^–1^ was consumed when *S*_0_ was raised above 1 g_*CS*_.L^–1^. This would mean that for concentrations below 1 g_*CS*_.L^–1^, external CO_2_ has been photosynthetically fixed at the expense of organic carbon consumption. This is reflected by the yield calculation. Mixotrophic growth can be described as the sum of heterotrophic growth (consumption of organic compounds) and autotrophic growth (fixation of CO_2_ through photosynthesis) (Abiusi et al., 2020). Microalgae biomass yields on substrate can come close to 1.0 during mixotrophic growth thanks to internal CO_2_ fixation. For example, Abiusi et al. (2020) reached a yield of 0.94 g_*CX*_.g_*CS*_^–1^ by cultivating *C. sorokiniana* on acetate in mixotrophy, which is consistent with the results obtained here for 2-5 g_*CS*_.L^–1^. Higher yields on the other hand indicate that extra atmospheric CO_2_ was fixed by the cells. Yields on substrate (*Y*_*X/S*_, g_*CX*_.g_*CS*_^–1^) were found to decrease linearly with the amount of substrate consumed from 1.9 down to 0.85 g_*CX*_.g_*CS*_^–1^ after which they remained constant (Figure 5A). The trend was independent of the type of substrate. Regarding butyrate, a yield of 1.92 g_*CX*_.g_*CS*_^–1^ was obtained when 0.4 g_*CS*_.L^–1^ butyrate was consumed, slightly higher than the 1.69 g_*CX*_.g_*CS*_^–1^ measured by Turon et al. (2015b). Little additional information can be retrieved about biomass yields on higher butyrate concentrations due to the inhibitory effects previously described. The results thus confirm previous observations made on acetate and provide new information regarding growth on butyrate.

To further illustrate the contribution of inorganic and organic carbon to yield, a mass balance analysis was performed on carbon (Figure 5B). For *S*_0_ between 0.5 and 1.0 g_C_.L^–1^, carbon from atmospheric CO_2_ accounted for 0.48 and 0.31 of the carbon in biomass, no matter the substrate nature. For higher *S*_0_, the amount of C-CO_2_ declined to negative values for both substrates. This means that cells fixed less atmospheric carbon at higher *S*_0_, leading to a reduced biomass yields. These calculations were made under the assumption that biomass composition of *C. sorokiniana* was close to the one of *C. reinhardtii.* Microalgae composition can indeed vary among species. However, both *C. sorokiniana* and *C. reinhardtii* being chlorophytes, it was suspected that they have a similar biomass composition. Some authors reported biomass composition of CH_1.82_O_0.42_N_0.18_ for *C. sorokiniana* (Kumar et al., 2014) cultivated in mixotrophy. Using this composition would lead to an error in estimation of 6.3%. Since (Kumar et al., 2014) did not provide the P composition, we used the one determined by Boyle and Morgan (2009) of CH_1.62_O_0.41_N_0.14_P_0.011_ as done in previous studies (Baroukh et al., 2017; Abiusi et al., 2020). Besides, the biomass composition was assumed to remain constant during the whole cultivation period. This assumption is probably verified during the exponential phase but may be incorrect once cells enter stationary phase. In this stage, if nutrients are limiting, cells may start accumulating carbonaceous storage compounds such as lipids or carbohydrates while degrading proteins to survive. Overall, this would reduce the amount of N and P per cell. To take this into account, calculations were done using reduced amount of N and P in the biomass. If the N and P content dramatically declined to 0.08 and 0.008, respectively, this would lead to an error of 4.3% in the carbon content estimation of 1 g biomass.

Altogether, the results suggest a competition between organic and inorganic substrate uptake. At VFA concentrations below 1 g_C_.L^–1^, it is possible that heterotrophic growth slowed down at some point while autotrophic growth could remain constant, implying that organic substrate consumption rate became slower than CO_2_ fixation rate. As cells are limited in the amount of total carbon they can assimilate (due to either pH, N, P or light), residual organic carbon remained unconsumed. As butyrate or acetate concentrations decreased in the medium, it may have dipped below the *K*_*S*_ (affinity constant) value of this algal strain for these substrates in these specific conditions. By raising the initial *S*_0_ above 2 g_C_.L^–1^ the residual organic concentration may stay above the critical *K*_*S*_ value for the whole cultivation duration. Heterotrophic growth could remain constant, and thus a greater organic substrate concentration could be consumed. In conclusion, *C. sorokiniana* could be cultivated in mixotrophy at high VFA concentration which resulted in higher substrate consumption. The observed decrease in biomass yield was due to a decrease in CO_2_ fixation rather than growth inhibition.

### Combined Effect of C:N:P and Buffer Concentration on Growth and Biomass Yields

**FIGURE 6:**
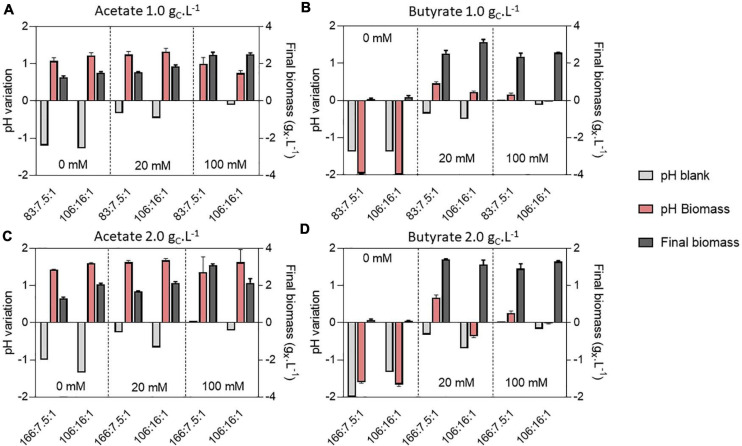
pH variation compared to the initial cultivation pH of 8.0 and final biomass (g_*X*_.L^–1^) (dark gray bars) obtained at the end of cultivation (7 days) on 1.0 or 2.0 g_C_.L^–1^ acetate **(A,C)** or 1.0 or 2.0 butyrate **(B,D)** in various buffer and C:N:P conditions. Variations were measured directly in the blank wells (light gray bars) or containing biomass (red bars). Blank data are given as a single value, while other data are given as mean ± standard deviation of 3 biological replicates.

**FIGURE 7:**
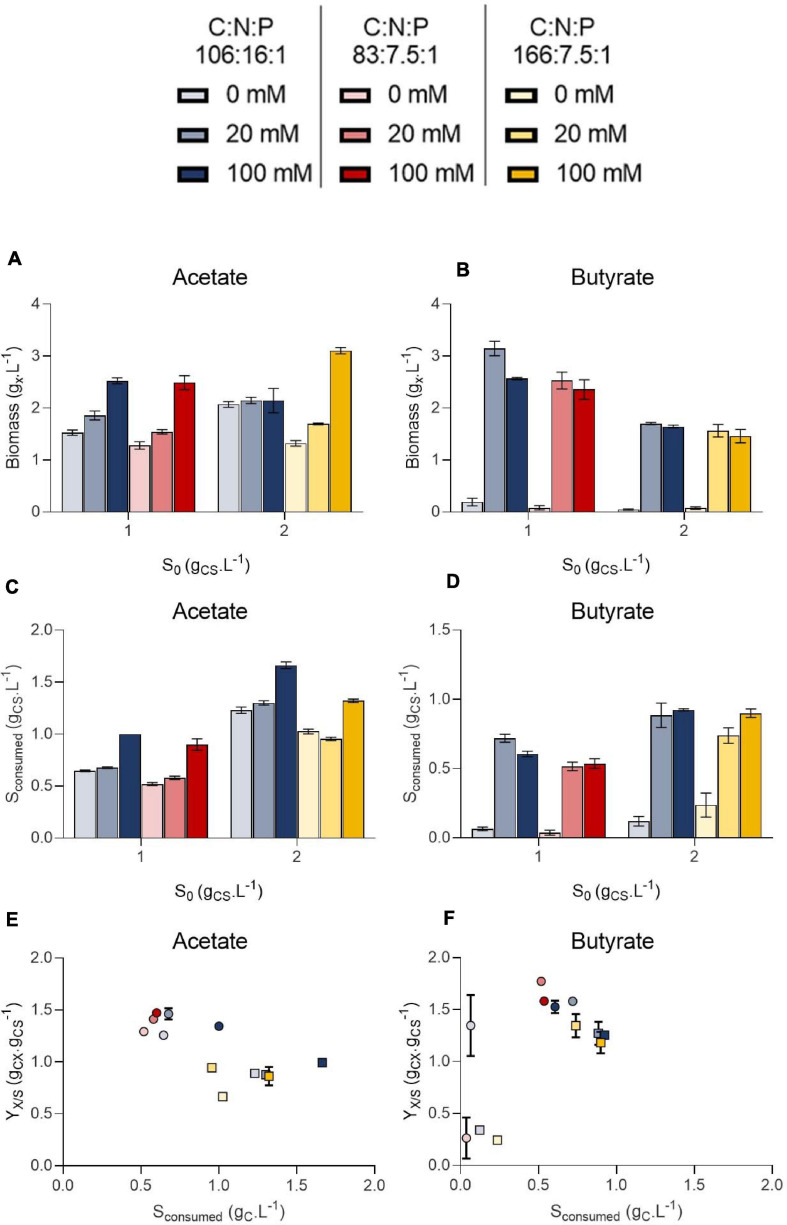
Final biomass (g_*X*_.L^–1^) obtained on 1.0 or 2.0 initial substrate (S_0_, g_C_.L^–1^) acetate **(A)** or butyrate **(B)**, amount of substrate consumed (g_C_.L^–1^) at the end of cultivation on 1.0 or 2.0 initial substrate (S_0_, g_C_.L^–1^) acetate **(C)** or butyrate **(D)** and biomass yields on acetate **(E)** or butyrate **(F)**. Color indicates C:N:P ratio 106:16:1 (blue), 83:7.5:1 (red) or 166:7.5:1 (yellow). Transparency indicates buffer capacity (0 mM for light, 20 mM for medium and 100 mM for dark color). The shape of the symbols indicate initial substrate concentration S_0_ (circles, 1 g_C_.L^–1^; squares, 2 g_C_.L^–1^). All data were derived from end point cultivation measurements (7 days in each case). Each point represents a mean ± standard deviation of 3 biological replicates.

To decipher the combined effect of nutrient (N, P) limitation and pH control on *C. sorokiniana* growth, substrate uptake and biomass yields, the microalgae were cultivated in microplates at 1.0 or 2.0 g_*CS*_.L^–1^ acetate or butyrate individually. For each concentration, two different C:N:P ratios were tested. Cells were cultivated on a C:N:P of 106:16:1, where all macroelements should be balanced, or on a C:N:P where nutrients (N or P) should limit growth, being 83:7.5:1 for 1.0 g_*CS*_.L^–1^ and 166:7.5:1 for 2.0 g_*CS*_.L^–1^. For each condition, cells were grown at various buffer capacities (0, 20 or 100 mM HEPES). Final pH measurements for each condition are reported on Figure 6 while final biomass concentrations, substrate consumption and biomass yields are reported on Figure 7.

**FIGURE 8:**
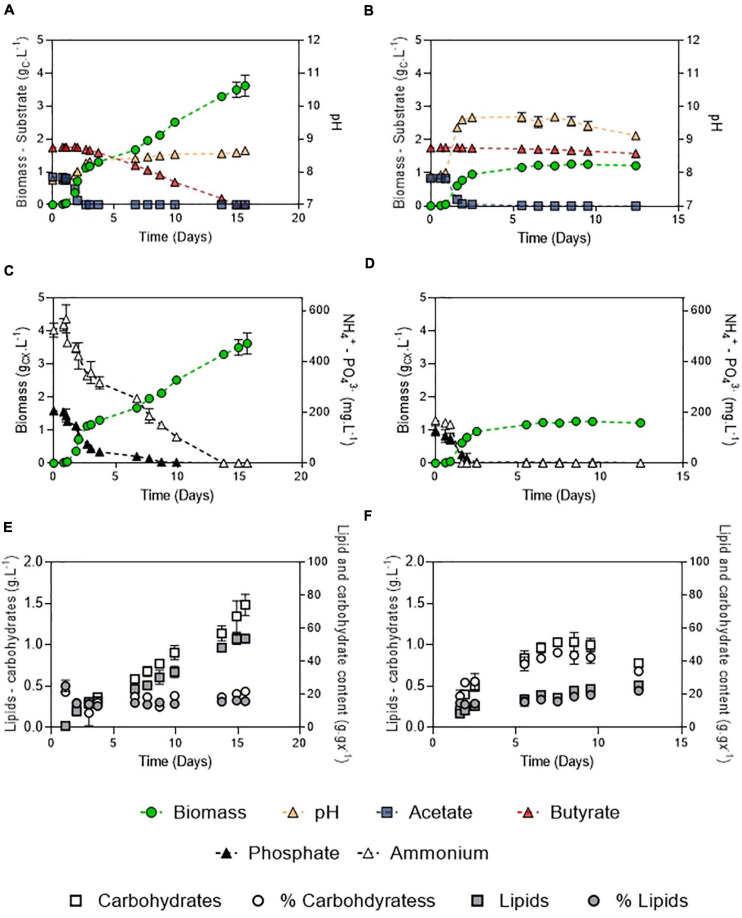
Growth curves, substrate dynamics and pH evolution of *C. sorokiniana* growing on 1:2 (g_C_.L^–1^) acetate:butyrate with either 100 mM buffer and 106:16:1 C:N:P **(A)** or 20 mM buffer and 250:7.5:1 C:N:P ratio **(B)**; nutrient evolution alongside biomass formation in obtained in the 100 – 106:16:1 **(C)** or the 20 – 250:7.5:1 culture **(D)**; carbohydrates and lipids concentration evolution alongside carbohydrates and lipid fraction in the biomass in the 100 – 106:16:1 **(E)** or 20 – 250:7.5:1 culture **(F)**. Each point represents a mean ± standard deviation of 3 biological replicates.

In presence of acetate, growth occurred in every condition and pH consistently rose to 9.0 – 9.5 (Figures 6A,C). In weakly buffered condition (0-20 mM), it can be supposed that pH rose to 10-11, as observed in Figure 3B. The main difference with the previous experiment resides in the cultivation duration, which lasted 7 days instead of 3. During microalgae cultivation, apart from organic acid consumption, pH may rise due to consumption of dissolved inorganic carbon. Following growth arrest, medium may have acidified due to solubilization and accumulation of atmospheric CO_2_ in the medium. The blank wells indeed indicate that without inoculum, and thus without organic acid or CO_2_ consumption, medium pH tended to decline. This observation was confirmed by measuring pH dynamics in flask (Figures 8A,B and Supplementary Figure 2A). It is likely that high buffer concentration (100 mM) impacted pH evolution dynamics, by slowing down the rate of pH increase or reducing the maximal value reached during cultivation. Indeed, increasing buffer capacity from 0-20 to 100 mM always resulted in increased or equal final biomass concentration even though final pH was not significantly different between each condition. However, C:N:P had little effect on final biomass concentration. For example, 1.5 g_*X*_.L^–1^ (C:N:P 106:16:1) and 1.3 g_*X*_.L^–1^ (C:N:P 83:7.5:1) of biomass were respectively obtained when cells were grown on 1.0 g_*CS*_.L^–1^ acetate without buffer. Increasing buffer capacity to 100 mM lead to an increase in final biomass by nearly two-fold to 2.6 g_*X*_.L^–1^. At 2.0 g_*CS*_.L^–1^ *S*_0_, 1.2 g_*X*_.L^–1^ were obtained in the 0 mM buffer - 166:7.5:1 C:N:P ratio. Increasing buffer capacity to 100 mM resulted in a two-fold increase, up to the same level obtained in the 0 mM buffer – 106:16:1 C:N:P condition. The biomass gain with higher buffer capacity was related to a higher substrate consumption (Figures 7C,D). For a given *S*_0_, substrate consumption always increased with buffer capacity, irrespective of C:N:P. The results are in accordance with the work of Shen et al. (2016), who observed consumption of acetate by *C. vulgaris* even during complete N and P starvation. Substrate consumption was also correlated to *S*_0_. As an illustration, cells appeared to be metabolically able to assimilate up to 1.0 g_C_.L^–1^ acetate even under the least favorable growth condition (0 mM buffer – C:N:P = 166:7.5:1) when initial acetate was set to 2 g_C_.L^–1^ (Figure 7C). When *S*_0_ decreased to 1.0 g_C_.L^–1^, in the same buffer and C:N:P condition, cells could only assimilate half that amount.

On the other hand, weak pH buffering was mandatory to prevent growth inhibition by butyrate (Figures 6B,D). In presence of butyrate, lack of pH buffering (0 mM) resulted in final pH dropping to 6.0 – 6.5. At these pH values, the concentration of undissociated acid BuOOH is 59.3 and 118.7 mg_C_.L^–1^ (1.0 or 2.0 g_*CS*_.L^–1^ *S*_0_) which is higher than the previously reported inhibiting concentrations (Lacroux et al., 2020). As a consequence of the slight acidification of the medium, BuOOH concentration reached an inhibitory threshold, preventing autotrophic and heterotrophic growth. However, buffering the medium with 20 mM HEPES was sufficient to prevent the pH drop. Cells were thus not inhibited and could grow. As observed in Section “Microplate Protocol Validation,” the pH did not rise as much as on acetate during butyrate cultivation, with a maximum pH variation of 0.5 unit in 20 mM buffer condition (Figures 6B,D). Thus, increasing buffer capacity from 20 to 100 mM did not further impact growth. In the case of butyrate, there was no significant difference in final biomass concentration between the various C:N:P ratios: the limiting factor was probably time. It is noted that in the case of real DFE, butyrate would be inevitably mixed with acetate, which fast consumption could prevent such acidification. However, in presence of acetate, pH control is needed to avoid extreme medium alkalization which would inhibit organic substrate uptake and growth.

Biomass yields obtained in each condition were plotted against the amount of substrate consumed for either acetate (Figure 7E) or butyrate (Figure 7F). These yields were poorly affected by either the amount of substrate consumed, buffer capacity or C:N:P. Instead, they mainly depended on the initial substrate concentration. Biomass yields clustered around 1.3-1.5 g_*CX*_.g_*CS*_^–1^ on 1.0 g_*CS*_.L^–1^ acetate, and increasing initial acetate to 2.0 g_*CS*_.L^–1^ resulted in decreased biomass yields by 26-48% depending on the condition. The same trend was observed on butyrate (disregarding 0 mM buffer points) with a maximum of 25% yield reduction. These results validate the observations made in the first section: heterotrophic growth slows down at low initial organic substrate concentration, leading to higher autotrophic contribution. Such competition effect is reflected by the decrease of the biomass yields when organic substrate concentration increased, indicating that the cells incorporated less external inorganic carbon. Consistently, Heifetz et al. (2000) showed that CO_2_ fixation by *C. reinhardtii* was reduced in presence of increasing acetate concentrations. Other authors consistently suggested that *C. reinhardtii* favored acetate assimilation over CO_2_ fixation (Chapman et al., 2015). Given that biomass yields also decreased when initial butyrate concentration increased, cells seem to also favor butyrate assimilation over CO_2_ fixation, as suggested by Liu et al. (2013a). The fact that increasing buffer capacity increase the amount of substrate consumed would thus imply that the specific growth rate on organic substrate is a direct function of pH. It is probable that VFA transportation in microalgae cells occurs via a monocarboxylate/proton transporter (MCT) as in other eukaryotes (Casal et al., 2008; Perez-Garcia et al., 2011). In this system, acetate in the anionic form is transported along with a proton (H^+^). In alkaline environment, transport might be too energy consuming for the cells to counteract the negative H^+^-gradient between the external medium and the near-neutral cytosolic pH.

### Biomass, Lipids, and Carbohydrates Production Potential of *C. sorokiniana*

After the initial screening of conditions in microplates, biomass production potential of *C. sorokiniana* was evaluated by cultivating the strain in Erlenmeyer flasks on a synthetic medium mimicking DFE composition. The carbon mass ratio of acetate:butyrate used was 1:2 (Figure 8) at a final concentration of 3 g_C_.L^–1^. This ratio and concentration are considered as representative of an average DFE (Moscoviz et al., 2018). Growth, substrates and pH dynamics were assessed under optimal conditions (100 mM buffer – 106:16:1 C:N:P) (Figures 8A-E) and compared to a condition where pH was weakly buffered and nutrients were limited (20 mM buffer, 250:7.5:1 C:N:P) (Figures 8B–F).

In both conditions, two growth stages were observed, corresponding to the two substrates. The diauxic behavior, in which acetate is taken up before butyrate, described by Liu et al. (2013b) and Turon et al. (2015a), was also observed here. First, the biomass quickly increased by consuming acetate (*μ* = 3.4 or 3.7 d^–1^). The presence of butyrate was not detrimental for growth on acetate but slightly inhibitory. Indeed, growth rates obtained in the mixture were reduced by about 5.1% compared to acetate alone in the same condition (20 mM buffer, N limitation, 3 g_C_.L^–1^ total concentration) (Figure 3C), which indicates that a slight inhibition occurred. In both conditions, acetate was consumed in less than 2 days (*Q_S_* = 0.51 or 0.48 g_*CS*_.L^–1^.d^–1^). Biomass yields on acetate were unaffected by the presence of butyrate, as consumption of acetate lead to a biomass yield of 1.01 g_*CX*_.g_*CS*_^–1^. The low buffer capacity induced a pH shift up to 9.6 while it remained stable around 8.3 in the culture at 100 mM buffer. Butyrate seemed to have an effect on pH homeostasis since in presence of butyrate, pH reached 9.6 instead of 11 on pure acetate (Figure 4B). These observations could also be due to the culture vessels (Erlenmeyers vs. microplates) that may favor gas transfer. However, data obtained on acetate alone in flasks (Supplementary Figure 2) show that pH indeed reached a value around 11-12 without butyrate, confirming the results and hypothesis made for the microplates experiments. No difference in either biomass productivity, biomass yield or substrate uptake rate was observed in this stage (Table 3). Ammonium consumption rate was slightly higher in the low buffer condition, suggesting that some stripping occurred. In the 250:7.5:1 C:N:P condition, N and P were completely exhausted at the end of this stage.

In a second stage, another growth phase or accumulation phase was observed depending on C:N:P. In the 106:16:1 condition, cells keep growing by consuming butyrate, which is exhausted after 10 days. They reach a final biomass concentration of 3.5 g_*CX*_.L^–1^. Consistently with the previous observations, biomass yield on butyrate was higher than on acetate (1.44 g_*CX*_.g_*CS*_^–1^). A 5-fold reduction in substrate consumption rate was concomitant to a reduction of the ammonium removal rate by the same order of magnitude. Phosphate depletion at day 10 did not stop cells from growing, probably owing to luxury uptake during the acetate phase. In these cultures, total lipids and carbohydrates content remained relatively stable during the whole cultivation period, accounting for 16.4 ± 0.2% and 21.9 ± 0.5 % (w/w) of the biomass after complete butyrate consumption, respectively (Figure 8E). On the other hand, in the 250:7.5:1 C:N:P cultures, pH elevation stopped the substrate uptake while nutrient limitation triggered carbohydrates accumulation. A doubling of the carbohydrates accumulation reaching up to 45.3 ± 1.1% was observed while lipid content remained around 15% (Figure 6F). After a prolonged limitation, carbohydrates content declined and lipid increased to 22.3 ± 0.8%. The related productivities are given in Table 4.

**TABLE 4:** Growth characteristics of *C. sorokiniana* cultivated in mixture acetate:butyrate (1:2 g_C_.L^–1^) with either 100 mM buffer and no nutrient limitation (100 mM – 106:16:1 C:N:P) or 20 mM buffer with nutrient limitation (20 – 250:7.5:1 C:N:P).

	100 mM – 106:16:1 C:N:P			20 mM – 250:7.5:1 C:N:P		
		
	Acetate		Butyrate	Acetate		Butyrate
*Xmax* (g_C_.L^–1^)	1.13 ± 0.10		2.37 ± 0.30	1.21 ± 0.01		0.10 ± 0.02
*Y* (g_*CX*_.g_*CS*_^–1^)	1.01 ± 0.04		1.44 ± 0.06	1.01 ± 0.02		NA
*μ* (d^–1^)	3.4 ± 0.1		0.13 ± 0.0	3.7 ± 0.1		NA
*Q*_*X*_ (g_*CX*_.L^–1^.d^–1^)	0.68 ± 0.06		0.20 ± 0.04	0.73 ± 0.01		NA
*Q*_*S*_ (g_*CS*_.L^–1^.d^–1^)	0.51 ± 0.00		0.10 ± 0.01	0.48 ± 0.01		0.03 ± 0.00
*Q*_*N*_ (mg_*NH4*_.L^–1^.d^–1^)	151.5 ± 34.7		35.5 ± 11.9	230.0 ± 13.9		NA
*Q*_*P*_ (mg_*PO4*_.L^–1^.d^–1^)	98.6 ± 17.8		9.4 ± 2.8	126.9 ± 3.0		NA
*Q*_*Lipid*_ (mg_*Lip*_.L^–1^.d^–1^)		75.6 ± 4.8			36.5 ± 1.2
*Q*_*carbohydrates*_ (mg_*Carbs*_.L^–1^.d^–1^)		99.4 ± 8.7			150.8 ± 1

*X_max_: maximum biomass (g_*X*_.L^–1^); *Y*: biomass yield on substrate (g_*CX*_.g_*CS*_^–1^); *μ*: growth rate (d^–1^); *Q:* production rate of biomass (*X*), consumption rate of substrate (*S*), nitrogen (*N*), or phosphate (*P*) or the production rate of lipid or carbohydrates. Growth parameters are calculated for either the acetate or butyrate stage. Values are given as mean ± standard deviation, *n = 3*. NA, Not Applicable.*

These results show that *C. sorokiniana* can consume VFAs entirely as long as pH is controlled and nutrients provided in adequate amounts. The biomass of *C. sorokiniana* is composed on average of 40% protein, 30–38% carbohydrate, and 18–22% lipid (Lizzul et al., 2018), which corresponds well to the values found here. Overall, in accordance with the literature, nutrient replete condition promoted biomass formation while nutrient limitation was found to trigger storage products accumulation. It has been shown that acetate feeding increased lipid content of cells compared to autotrophy (Cecchin et al., 2018). The main explanation of this finding was that in mixotrophic cells the acetyl-CoA pool would be considerably increased. Acetate is a simple substrate necessitating only one or two activation steps at the expense of one ATP molecule to produce acetyl-CoA. It is assumed to be produced in all main cellular compartments and can directly enter central carbon metabolism at the level of for example the glyoxylate cycle and TCA cycle, fatty acid synthesis for lipid production as well as starch production via gluconeogenesis (Smith and Gilmour, 2018). As butyrate degradation should also ultimately lead to acetyl-CoA, feeding cells with this substrate may also boost their lipid content. However, *C. sorokiniana* was found to first accumulate carbohydrates as short-term energy storage products, which were subsequently degraded into lipids after a prolonged starvation period. Such temporal accumulation pattern was already described by Li et al. (2015) when cultivating *C. sorokiniana* on glucose. In terms of lipid productivity, increasing biomass production rate, through appropriate C:N:P, is thus superior to using nutrient limitation strategy, since this triggers lipid accumulation only after long-term starvation or severe nutrient limitation. As a comparison example, Patel et al. (2021) cultivated *C. sorokiniana* in heterotrophy on a DFE permeate containing 2.8 g.L^–1^ acetate, 1.43 g.L^–1^ propionate, 1.41 g.L^–1^ butyrate, 0.25 g.L^–1^ valerate and 4.78 g.L^–1^ caproate. The strain was cultivated under mass C:N ratio 20 and 60. Although *C. sorokiniana* accumulated up to 33.8% of its DW as lipids under the most severe limitation condition (mass C:N 60, corresponding to a molar C:N ratio of 70), a very low biomass yield was obtained (0.07 g_*X*_.g_*S*_^–1^), resulting in a low lipid productivity (0.045 g_*Lip*_.L^–1^.d^–1^). During the active growth phase of batch cultures, or in continuous conditions, the maximum productivity *Q*_*X*_ is a combination of growth rate and carbon reserve content, which are basically inversely related (Yang et al., 2018). The higher biomass productivity will result in biomass less concentrated in lipids and carbohydrates, which is less preferable for extraction processes since larger volumes need to be processed. This trade-off between productivity and lipid content has been extensively discussed in the case of autotrophic cultivations (Adams et al., 2013) and mixotrophic regimes are no exception. In synthetic conditions, improvement of lipid content can be done via different strategies, such as response surface methodology (Khanra et al., 2020). This kind of optimization may however be more challenging in the case of cultivation on DFE, since C:N:P content cannot be fully controlled, as it mostly depends on the initial feed in the fermenter. In this study, relatively low concentrations, ranging from 105 to 550 mg.L^–1^ of N-NH_4_, were used. N content is not always monitored in DFEs, but as a comparison, N-NH_4_ can reach concentrations higher than 5000 mg.L^–1^ N-NH_4_ in anaerobic digestion effluents (Yenigün and Demirel, 2013). Such concentrations could be inhibitory to microalgae growth, since at pH 9.2 and above, free ammonia become the dominant species. Although *C. sorokiniana* can tolerate ammonia concentration up to 96.3 mg.L^–1^ NH_3_ (Rossi et al., 2020), this would in any case exclude a N limitation strategy for lipid accumulation. The effect of high NH_4_ concentration on the mixotrophic growth of microalgae on VFA, out of the scope of the current study, should be thus further evaluated.

## Conclusion

Very little research has been done on DFE-algae systems so far and therefore their potential is not yet well known. This study aimed at providing insights in *C. sorokiniana* physiology in presence of acetate and butyrate, the two main components of DFEs. A detailed microplate protocol was thus designed, enabling analysis of multiple parameters simultaneously. A first step in improving the coupled process is to allow cultivation of microalgae on concentrated effluent. To tackle this issue, the microalgae were cultivated at an initial pH value of 8, which minimized the inhibitory undissociated acid concentration. Acetate and butyrate concentration up to 12.5 g.L^–1^ and 8.5 g.L^–1^ respectively were thus not inhibitory to microalgae growth. If growth rates were slightly impacted by increasing VFA concentrations, mixotrophic biomass yields decreased substantially indicating a shift towards heterotrophy. If cultivation on concentrated effluent seems feasible, other challenges need to be addressed. First, the massive pH increase due to VFA assimilation implies a tight pH control for complete VFA assimilation. In this study, pH was controlled using 100 mM buffer. Use of buffer at industrial scale is excluded and other forms of pH control should be envisaged. As DFE are typically in the range of pH 5-6, a first possibility would be to control the feeding according to pH elevation. However, due to diauxic assimilation of acetate and butyrate, such a strategy could lead to accumulation of butyrate. Alternatively, pH control can be achieved by bubbling the CO_2_-riched biogas from the fermentation tank into the microalgae cultivation. This would allow simultaneous biogas purification from CO_2_, a necessary post-treatment in order to obtain pure H_2_, and potentially increase mixotrophic yields. This strategy would however require deeper knowledge of the mixotrophic regulation in presence of both carbon sources. Although C:N:P ratio had little effect on biomass yields, it triggered accumulation of carbohydrates.

Controlling the C:N:P of DFEs may be challenging as the levels of these nutrients will depend on the feedstock and the total solids in the bioreactor that served for DF and can thus vary widely. *C. sorokiniana* may not be well suited for lipid production as the strain was found to preferentially accumulate carbohydrates in short term nutrient limitation. This trait could however be advantageously used to produce a carbohydrate-rich biomass serving as feedstock for subsequent fermentation into H_2_. In addition, different environmental isolates may very well prove more adapted to the production of lipids since important differences in physiology exist within the same species.

## Data Availability Statement

The original contributions presented in the study are included in the article/Supplementary Material, further inquiries can be directed to the corresponding author/s.

## Author Contributions

JL contributed to the conceptualization and performed the experiments, data acquisition, data curation, formal analysis, and writing of the original draft. JS and RL designed the original experimental plan and contributed to the formal analysis, supervision, funding acquisition, validation, and review and editing of the original draft. J-PS, ET, and NB contributed to the formal analysis, supervision, funding acquisition, validation, and review and editing of the original draft. All authors approved the final version of the manuscript.

## Conflict of Interest

The authors declare that the research was conducted in the absence of any commercial or financial relationships that could be construed as a potential conflict of interest.
