# Supplementary material for: Mixotrophic Growth of Chlorella sorokiniana on Acetate and Butyrate: Interplay Between Substrate, C:N Ratio and pH
Source: Front Microbiol. 2021 Jul 2;12:703614. doi: 10.3389/fmicb.2021.703614 (PMC8283676; doi:10.3389/fmicb.2021.703614)
Supplement: Supplementary file 1 [file Data_Sheet_1.docx]

Supplementary Material

# Supplementary Information

## Microplates protocol validation

Microplate cultivation provides an efficient way to screen multiple parameters at once and gather biomass growth dynamics data. However, their use presents several drawbacks that need to be taken into account to ensure accuracy of measurements. The following paragraphs aim at presenting the methods used to circumvent the biases induced by the use of microplates. A schematic representation of the microplate is shown in fig. SI1.


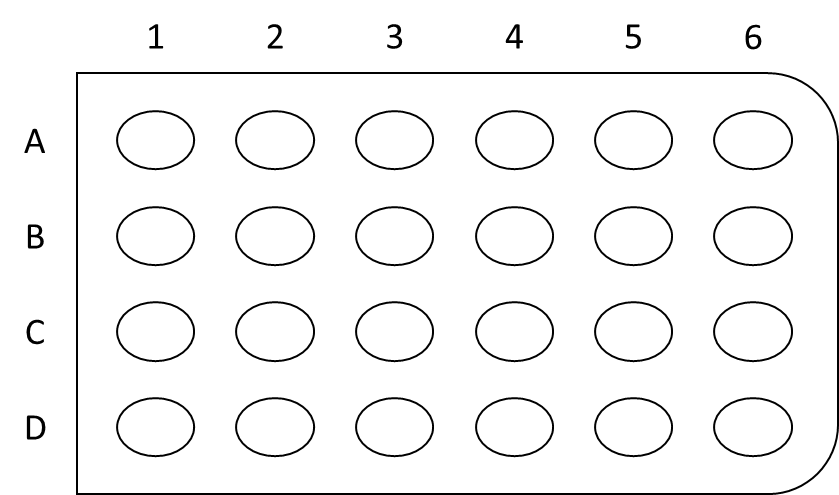


**Figure SI 1 :** Schematic drawing of a 24-well microplate

## Evaporation

First, due to the small volume used (up to 1.5 mL in 24-wells plates), water evaporation is a major concern and cannot be considered negligible, especially in the outer wells. To answer this issue, we measured the water evaporation rate in each well in the growth conditions of our incubator (25°C, 100 rpm, 100 µE light intensity). The 24 wells of a plate were filled with 1.5 mL water buffered to pH 8.0. The corresponding mass (of an individual well) was measured. Twice per day, the mass of each well was measured. This was done by pipetting the content of a well, measuring the corresponding mass and putting back the liquid in the well. The measurements were done for 3 different plates. The kinetic of water evaporation in each separate well could thus be determined (Table S1). Each well presented a distinct kinetic. It should be noted that in some cases, the linearity was poor (R² < 0.9) and that great discrepancies are observed between each separate plate. In any cases, evaporation was high, up to 315 µL/d in some wells. To circumvent this issue, another test was performed. The evaporation was controlled by wrapping each microplate in individual plastic sachet. Light intensity measured in the sachet was found not different to the one measured without. The measurements of water evaporation were repeated using the same protocol as described above. The measurements were done for 6 different microplates and two distinct repetitions were done.

**Table SI 2:** Water evaporation rate (µL/d) of the 24 wells for three different plates. The water evaporation rate was determined by plotting the evolution of mass per day in each well.

| Wells # | Plate A | | Plate B | | Plate C | |
| --- | --- | --- | --- | --- | --- | --- |
|  | µL/d | r² | µL/d | r² | µL/d | r² |
| A1 | 288.41 | 0.97 | 72.25 | 0.66 | 68.46 | 0.98 |
| A2 | 164.65 | 0.95 | 40.82 | 0.97 | 41.51 | 0.95 |
| A3 | 128.34 | 0.96 | 37.44 | 0.98 | 24.93 | 0.93 |
| A4 | 99.45 | 0.97 | 39.45 | 0.99 | 26.39 | 0.81 |
| A5 | 94.03 | 0.84 | 62.64 | 0.98 | 41.48 | 0.66 |
| A6 | 144.58 | 0.39 | 185.00 | 0.96 | 111.89 | 0.19 |
| B1 | 315.10 | 0.97 | 96.60 | 0.36 | 149.88 | 0.95 |
| B2 | 162.48 | 0.97 | 45.29 | 0.92 | 61.07 | 0.92 |
| B3 | 122.81 | 0.96 | 37.95 | 0.98 | 34.94 | 0.90 |
| B4 | 106.07 | 0.92 | 43.99 | 0.99 | 25.30 | 0.99 |
| B5 | 103.17 | 0.59 | 74.58 | 0.97 | 38.72 | 0.68 |
| B6 | 162.12 | 0.14 | 234.33 | 0.96 | 105.48 | 0.25 |
| C1 | 311.11 | 0.97 | 107.03 | 0.31 | 257.76 | 0.95 |
| C2 | 155.86 | 0.97 | 47.34 | 0.91 | 98.67 | 0.96 |
| C3 | 118.95 | 0.97 | 38.31 | 0.99 | 52.18 | 0.97 |
| C4 | 105.84 | 0.89 | 40.76 | 1.00 | 35.03 | 0.98 |
| C5 | 106.46 | 0.54 | 72.93 | 0.97 | 38.32 | 0.80 |
| C6 | 165.78 | 0.06 | 225.12 | 0.96 | 69.96 | 0.60 |
| D1 | 256.30 | 0.97 | 92.62 | 0.25 | 302.16 | 0.96 |
| D2 | 127.56 | 0.98 | 45.66 | 0.84 | 110.58 | 0.96 |
| D3 | 102.21 | 0.96 | 36.84 | 1.00 | 51.84 | 0.98 |
| D4 | 103.09 | 0.84 | 41.49 | 0.99 | 39.78 | 0.98 |
| D5 | 108.82 | 0.57 | 58.37 | 0.98 | 38.31 | 0.97 |
| D6 | 158.57 | 0.27 | 161.34 | 0.96 | 52.63 | 0.99 |

The use of the plastic sachet presented two advantages. First, a greater linearity was observed in each well which enabled more accurate measurements. The water evaporation rates are besides much more homogenous in each well. Statistical tests show indeed that evaporation rates between each well were not significantly different. In the same manner, each plate was not different from another one. The results were confirmed by the second repetition. Secondly, water evaporation rates were greatly reduced by about 2 to 5 fold, with an average evaporation rate (calculated based on the average evaporation rate of each well and the 2 repetitions) of 25.8 µL/d.

**Table SI 3:** Water evaporation rate (µL/d) when plates are wrapped in plastic sachets. Each measurement represents the mean and standard deviation of 6 different plates.

| Wells # | Repetition 1 | | Repetition 2 | |
| --- | --- | --- | --- | --- |
|  | µL/d | | µL/d | |
|  | mean | SD | mean | SD |
| A1 | 25.17 | 5.62 | 21.11 | 7.49 |
| A2 | 27.46 | 4.86 | 23.28 | 6.09 |
| A3 | 28.83 | 6.28 | 24.13 | 5.82 |
| A4 | 28.82 | 4.62 | 24.44 | 4.43 |
| A5 | 27.91 | 2.95 | 25.15 | 3.76 |
| A6 | 28.48 | 3.34 | 25.84 | 3.10 |
| B1 | 27.78 | 6.13 | 20.52 | 9.30 |
| B2 | 27.10 | 5.60 | 23.06 | 6.17 |
| B3 | 27.19 | 2.56 | 25.45 | 4.50 |
| B4 | 24.41 | 4.96 | 26.68 | 3.52 |
| B5 | 24.33 | 6.30 | 27.84 | 3.92 |
| B6 | 26.42 | 4.98 | 25.58 | 3.29 |
| C1 | 23.40 | 7.64 | 21.18 | 8.97 |
| C2 | 26.07 | 6.35 | 24.40 | 7.65 |
| C3 | 23.46 | 5.57 | 26.95 | 4.64 |
| C4 | 25.43 | 5.74 | 28.06 | 3.59 |
| C5 | 23.94 | 3.46 | 27.59 | 4.10 |
| C6 | 26.44 | 3.08 | 27.23 | 4.24 |
| D1 | 25.65 | 3.88 | 22.70 | 8.44 |
| D2 | 27.84 | 2.37 | 25.10 | 6.58 |
| D3 | 26.96 | 7.83 | 26.41 | 6.08 |
| D4 | 24.70 | 2.14 | 27.30 | 4.79 |
| D5 | 26.42 | 5.62 | 28.05 | 3.84 |
| D6 | 28.03 | 4.54 | 27.71 | 3.56 |

If this evaporation rate is low and not significant over short cultivation period, it becomes relevant when cultivation time are extended (as they are in our experiments). Thus, sterile distilled water was added each day in each well to compensate the water loss. The amount of water added was calculated using the mean evaporation rate of 25.8 µL/d.

It should be noted that microplates were removed from the plastic sachet before measurement. Air in the sachet was thus refreshed between each measurement (2 to 4 times a day).

## Condensation

Water evaporation is associated with condensation on the lid of the microplate. Excess condensation can prevent light from penetrating into the wells as well as giving overestimated optical density (OD) measurements. To prevent this, lids were aseptically wiped using KimWipe® papers before each measurement.

## Agitation and homogenisation

Agitation inside a well is not ensured due to its low volume. As such, cells tend to aggregate at the centre of the well and are not properly dispersed. Thus, light intensity may not be homogenous for all cells and gas transfer rate is not optimal. Besides, when optical density is measure, the aggregation of cells can lead to overestimation of the actual value. To circumvent this issue, wells were regularly homogenised by mixing with a sterile pipette. This was done before each measurement and at least 4 times a day. This ensured proper homogenisation before actual OD measurement. This technique does not tackle the low gas transfer issue. However, since a mixotrophic mode of growth was studied, this bias should not interfere heavily with our results (see section 1.5).

## Optical density saturation

OD measurement is a convenient way to estimate biomass growth using proper calibration curves. However, the linearity range of such calibration curves need to be properly determined. In the case of the Beer-Lambert law, it is well known that above certain concentration, optical density measurements are not valid because of the signal saturation. Thus, too high OD densities should be avoided using a proper sample dilution to reach an appropriate OD value, typically below 0.5 – 0.6. Such dilution can easily be done when using large culture vessels: samples can be withdrawn and diluted at will. In the case of microplates, samples are excluded (which is the basis of the technique). OD are always measured in the plate. As biomass increases, signal may become saturated without any possibility to dilute the culture. This holds especially true in the later stage of the growth curve. Thus, a legitimate question would be whether the plateau observed in the late growth stage is due to the cells reaching stationary phase or to a signal saturation. To answer this question, the OD of concentrated microalgae suspension was measured with or without dilution. Briefly, *C. sorokiniana* was cultivated in an Erlenmeyer flask to produce a dense fully grown culture. The OD_750_ of such a culture was measured to be 2.05 using a standard cuvette spectrophotometer. The culture was diluted several times. Above OD_750_ 0.5, the calculated OD_750_ based on the raw OD_750_ for the cuvette reader declines greatly, with a signal loss of up to 51%. This indicates that saturation occured. In the case of the microplate reader, OD_750_ remained constant for each dilution tested. Signal loss between undiluted and diluted sample did not exceed 3.8%. We concluded that even if a slight saturation occurs during OD_750_ measurement, this should not prevent accurate prediction of biomass evolution.

# Supplementary Figures and Tables

## Supplementary Figures


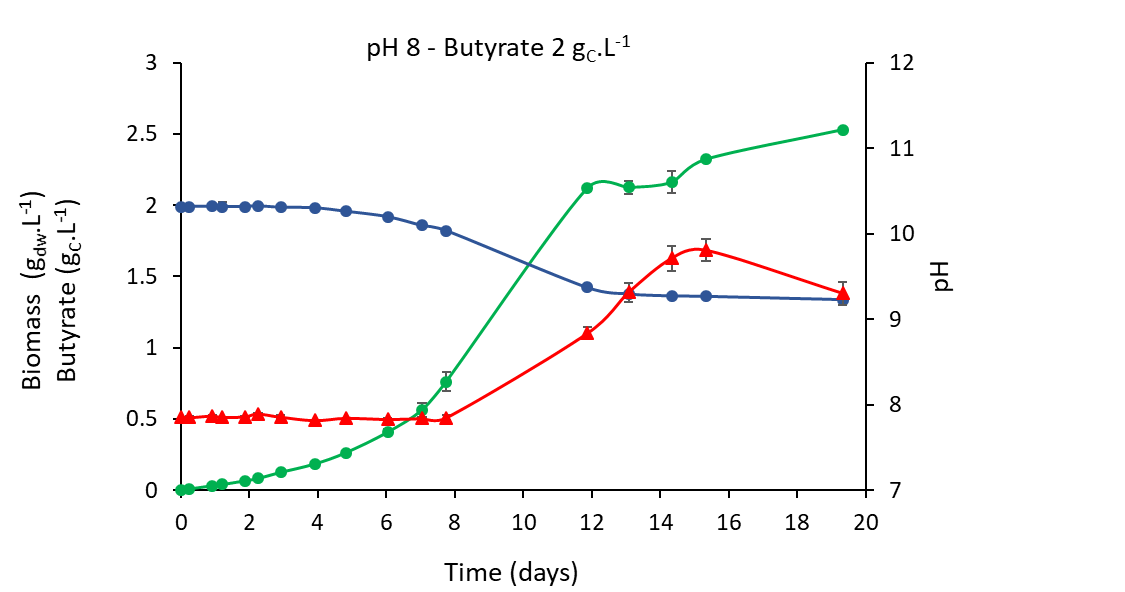

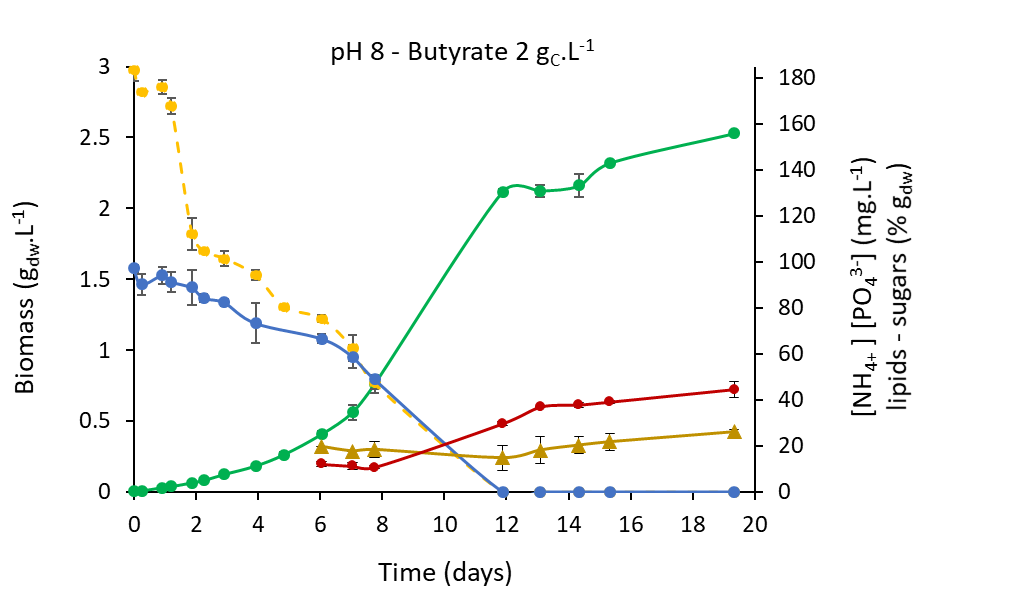

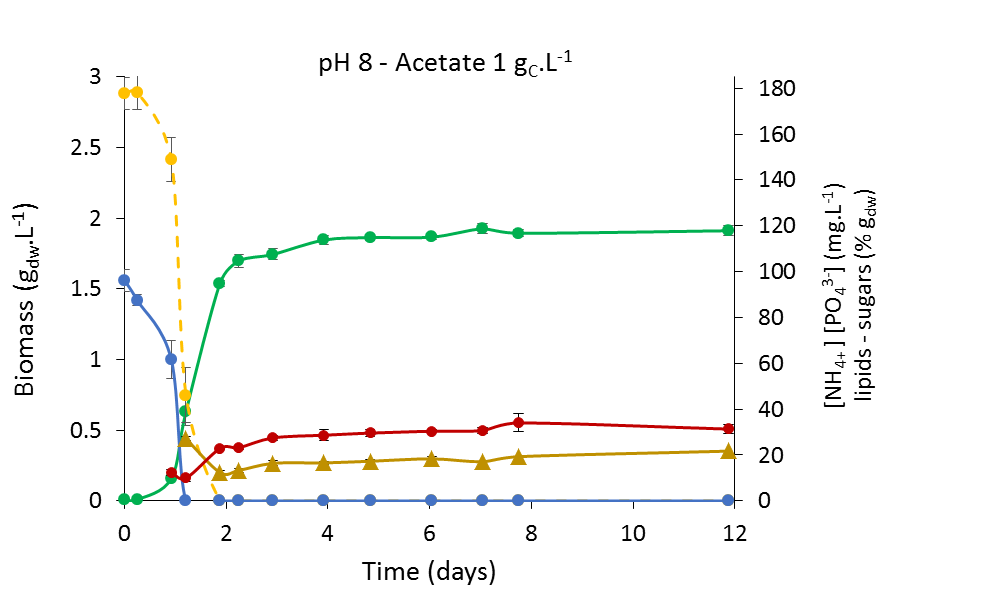


**B**


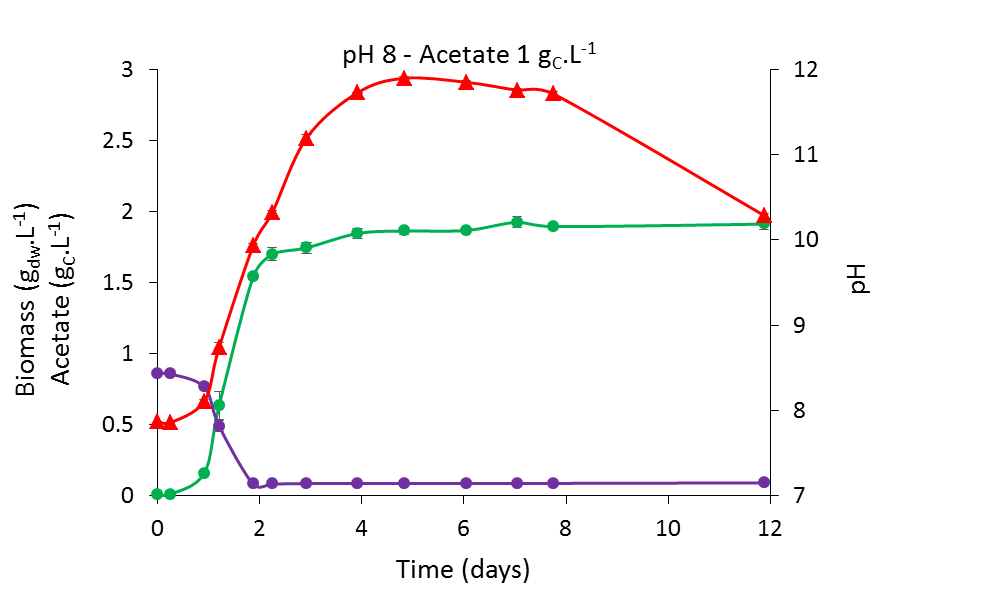


**A**


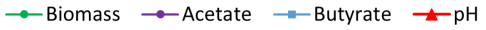

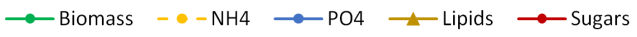


**C**

**D**

**Figure SI 2:** Evolution of biomass, substrate, nutrients (NH_4_^+^, PO4_3_^2-^), pH, lipids and carbohydrates of *C. sorokiniana* cultured on 1 g_C_.L^-1^ acetate **(A, B)** with a 83:7.5:1 C:N:P or 2 g_C_.L^-1^ butyrate with a 166:7.5:1 C:N:P **(C, D)**. Buffer capacity was in both cases set to 20 mM.
